# Suppression of Post-Ischemic Cardiac Remodelling and Inflammatory Response by a Novel Sphingolipid Modifier, CIN038

**DOI:** 10.3390/ijms27135776

**Published:** 2026-06-26

**Authors:** Bing H. Wang, Feby Savira, Xin Xiong, Daniel D. Donner, Helen Kiriazis, Aascha Brown, Li Huang, Natalie Mellet, Kevin Huynh, Peter J. Meikle, Darren Creek, Christopher Reid, Bernard L. Flynn, David M. Kaye, Danny Liew, Ruth R. Magaye

**Affiliations:** 1 Heart Failure Research Group, Baker Heart and Diabetes Institute, Melbourne 3004, Australia; bing.wang@baker.edu.au (B.H.W.);; 2 Monash Alfred Baker Centre for Cardiovascular Research, School of Translational Medicine, Monash University, Melbourne 3004, Australia; 3 Biomarker Discovery, Baker Heart and Diabetes Institute, Melbourne 3004, Australia; 4 Centre for Cardiovascular Research in Therapeutics, School of Public Health and Preventive Medicine, Monash University, Melbourne 3004, Australia; 5 School of Health and Social Development, Faculty of Health, Deakin University, Melbourne 3125, Australia; 6 Translational Cardiology Centre, Baker Heart and Diabetes Institute, Melbourne 3004, Australia; 7 Metabolomics Research Group, Baker Heart and Diabetes Institute, Melbourne 3004, Australia; 8 Monash Institute of Pharmaceutical Sciences, Monash University, Melbourne 3052, Australia; 9 School of Public Health, Curtin University, Perth 6102, Australia; 10 Faculty of Medicine, University of Adelaide, Adelaide 5000, Australia

**Keywords:** cardiac fibrosis, dihydroceramide desaturase 1, inflammation, interleukin-6, ischemia–reperfusion injury, ether phospholipid

## Abstract

In patients with myocardial infarction (MI), the level of sphingolipids, such as ceramide (Cer), is elevated and is associated with an increased risk of progression towards heart failure (HF). Dihydroceramide desaturase 1 (DES1) catalyses the conversion of dihydroceramide (dhCer) into Cer in the de novo sphingolipid pathway. While pharmacological inhibition of DES1 has shown therapeutic benefits in metabolic disease and cancer models, its role in cardiac remodelling remains unclear. This study aimed to determine whether pharmacological inhibition of DES1 using the novel compound, CIN038, attenuates cardiac remodelling following ischemia–reperfusion (I/R) injury. Three-month-old male C57Bl/6 mice underwent I/R or sham surgery (n = 8) and were treated with vehicle or CIN038 (50 mg/kg/day, i.p.) for 28 days. Cardiac function, molecular changes, and lipid profiles in circulation and liver were assessed at the endpoint. CIN038 reduced infarct size and cardiac myocyte hypertrophy compared to the I/R + vehicle group. Profibrotic signalling was reduced in the infarcted hearts, as evidenced by reduced expression of *Col1a1*, *Col3a1*, and *Tgfb* mRNA and decreased levels of α-SMA and TGFβ1 protein expression. Inflammatory signalling was attenuated with reduced ERK and NFkB phosphorylation and suppression of Il-6-STAT axis. Despite these structural and molecular improvements, no changes were observed in cardiac function. Lipidomic analysis revealed selective alterations in circulating and hepatic lipid species, including plasmalogen phosphatidylethanolamines and ether-linked triglycerides, suggesting modulation of lipid metabolism. Collectively, these findings indicate that CIN038 attenuates post-ischemic cardiac remodelling by suppressing inflammatory and profibrotic signalling, highlighting DES1 as a potential therapeutic target following MI.

## 1. Introduction

Interest in sphingolipids in the pathophysiology of cardiovascular diseases (CVD) has gained momentum due to their altered levels in clinical and animal studies [1,2,3]. The bioactive sphingolipids regulate multiple cellular processes, including apoptosis, inflammation, and fibrosis, suggesting their potential role in myocardial injury [4]. Within the de novo sphingolipid pathway, the enzyme dihydroceramide 1 (DES1) acts as a gatekeeper, catalysing the insertion of a 4,5-trans-double bond into dihydroceramide (dhCer) to form ceramide (Cer) [5,6,7]. Cer can be regarded as a metabolic hub, acting as a substrate for complex sphingolipids and sphingosine 1 phosphate (S1P) from sphingosine (Sph). Cer levels have been found to be elevated in the plasma of patients with coronary artery disease (CAD) and cardiac tissues of small animal models of heart failure (HF) [8,9]. Increased Cer in circulation is also associated with increased risk of HF, potentially due to its pro-apoptotic and pro-inflammatory effects [10,11,12]. Further downstream of Cer, the sphingolipid S1P is a bioactive molecule capable of activating cellular signalling pathways through its cell surface receptors (S1PR1-5), conferring both cardioprotective and injurious effects depending on the aetiology and the subtype of receptor being activated [13,14]. S1P has been implicated in fibroblast activation and extracellular matrix (ECM) remodelling through the sphingosine 1 kinase (Sphk1)/S1P axis, which can amplify transforming growth factor-β (TGFβ)-mediated profibrotic signalling [15,16,17,18]. These findings highlight the importance of sphingolipid metabolism in regulating inflammatory and fibrotic pathways that contribute to adverse cardiac remodelling following myocardial infarction (MI).

Targeting DES1 to modulate sphingolipid metabolism has emerged as a potential therapeutic strategy in cancer and hepatic diseases [19,20]. For example, inhibition of DES1 by the synthetic retinoic acid, fenretinide, reduces Cer and raises dhCer levels, resulting in increased apoptosis and reduced proliferation and angiogenesis in various cancers [5,21,22]. In terms of metabolic disorders, such as diabetes mellitus, DES1 inhibition increases insulin sensitivity and reduces adipocyte differentiation and senescence [5,23]. Despite these demonstrations of the therapeutic potential of DES1 inhibition, the use of non-specific inhibitors has led to inconsistent results and limited mechanistic insight. Furthermore, the effects of DES1 inhibition in the context of CVDs, including cardiac remodelling in MI, have not been explored.

Although DES1 specifically regulates sphingolipid desaturation, broader lipid metabolic changes may also occur following its inhibition due to the extensive crosstalk between lipid pathways. For example, alterations in sphingolipid metabolism can influence homeostasis of other lipids such as glycerophospholipids, diacylglycerols (DAGs), and triglycerides (TGs) [4,24,25,26].

We investigated the therapeutic potential of a novel sphingolipid-modifying agent, CIN038, in a mouse model of ischemia–reperfusion (I/R) injury. CIN038 is a synthetic sphingolipid-derived specific inhibitor of the DES1 enzyme with an IC_50_ of 0.55 µM as published by Aurelio et al. [27]. It is compound number 38 in the article, with the chemical name 4-((5-(4-trifluoromethyl) phenyl)-1,3,4-oxadiazol-2-yl) amino)-phenol.

## 2. Results

### 2.1. CIN038 Treatment Reduced Infarct Size and Myocyte Hypertrophy

**Figure 1:**
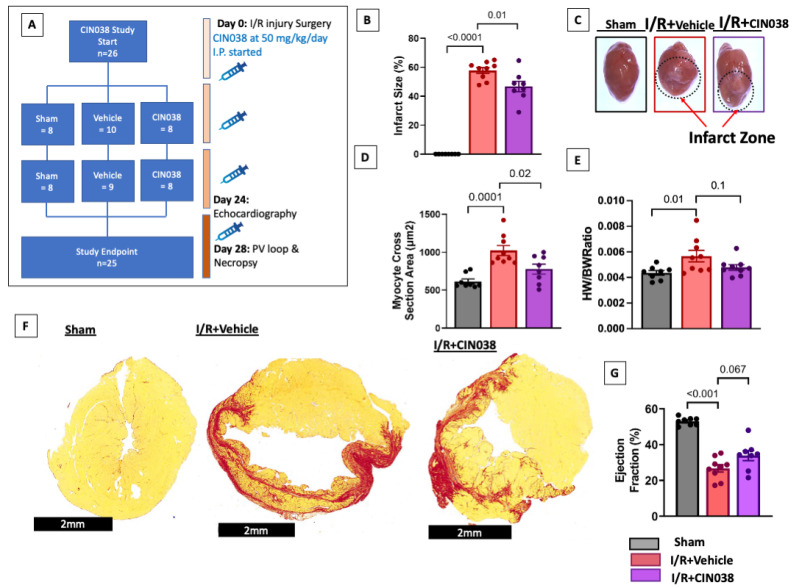
Effects of 50 mg/kg/day treatment with CIN038 on ischemia–reperfusion (I/R) injury-induced heart failure in C57Bl/6 male mice. (**A**) A total of 25 mice were included in this study: sham n = 8, ischemia–reperfusion (I/R) + vehicle n = 9, and I/R + CIN038 n = 8. (**B**,**C**) Infarct size (%), (**D**) cardiac myocyte section area, um2, and (**E**) heart weight to body weight (HW/BW) ratio. (**F**) Photographs of the heart sections showing the area of infarct (red). (**G**) Ejection fraction (%). *p* < 0.05 is considered significant. Data are presented as ± SEM and analysed using one-way ANOVA with Dunnett’s post hoc test.

We first looked at the effects of CIN038 on I/R injury-induced changes in cardiac structure and function. CIN038 treatment at 50 mg/kg/day over 4 weeks in mice (Figure 1A) significantly reduced the infarct size (%, *p* = 0.009), myocyte cross-sectional area (*p* = 0.02), and the elevated heart weight/body weight ratio (*p* = 0.04) compared to the I/R + vehicle group (Figure 1B–F). The infarct size was determined from pico sirius red (PSR)stained heart sections (Figure 1F). Ejection fraction (EF%) recovery was not significant in the I/R + CIN038 vs. the I/R + vehicle group (32.8 ± 2.9 vs. 29.6 ± 3.7%, *p* = 0.07) (Figure 1G). Both infarct size and EF% (53.0 ± 0.8 vs. 29.6 ± 3.7%) were significantly impacted by 40 min I/R injury in the I/R + vehicle group vs. sham (*p* < 0.0001). The marked increases in other echocardiographic measures of LV volume and area at systole (LVVs, LVAs) and diastole (LVVd; LVAd) in the I/R + vehicle group were not changed by CIN038 treatment (Supplementary Table S2). CIN038 treatment also had minimal effect on cardiac hemodynamic measurements on day 28 that were impacted by I/R injury (Supplementary Table S3).

### 2.2. CIN038 Treatment Reduced Fibrosis Markers Linked to the Tgfb Pathway

**Figure 2:**
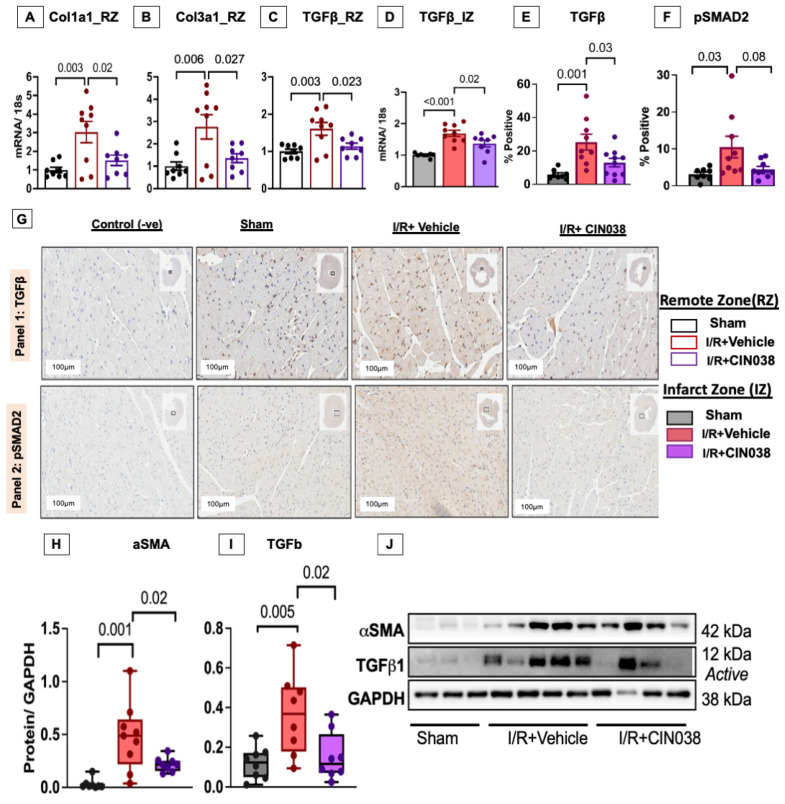
CIN038 reduced *Col1a1*, *Col3a1*, and *TGFβ* mRNA in the remote and infarcted zones differently. CIN038 treatment reduced (**A**–**C**) *Col1a1*, *Col3a1*, and *TGFβ* mRNA expression in the remote zone C (RZ) of the heart. *TGFβ* (**D**) mRNA in the infarct zone (IZ) and (**E**) cellular expression were reduced by CIN038. (**F**) Smad2 phosphoprotein-expressing cells were reduced. (**G**) Images of histochemical staining of TGFβ, Panel 1, and pSMAD2, Panel 2. Western blot analysis of (**H**) aSMA and (**I**) TGFb. (**J**) Representative blots. Sham n = 8, ischemia–reperfusion (I/R) + vehicle n = 9, and I/R + CIN038 n = 8. Data are presented as ± SEM and analysed using one-way ANOVA with Dunnett’s post hoc test. *p* < 0.05 is considered significant.

Since the pathophysiological and molecular changes at the site of injury, the infarct zone (IZ), and those at sites away from the injury, the remote zone (RZ) are different, we investigated the molecular changes within these areas separately after CIN038 treatment. Daily IP injections of CIN038 treatments in mice with I/R injury led to marked reductions in mRNA expressions of *Col1a1* (*p* = 0.02), *Col3a1* (*p* = 0.04), and *Tgfb* (*p* = 0.02) in the RZ compared to the I/R + vehicle group (Figure 2A–C). Within the site of injury, only *Tgfb* mRNA (*p* = 0.02, Figure 2D) was significantly reduced by CIN038 treatment vs. the vehicle group. These were all significantly elevated in the vehicle vs. sham groups. This reduction was also supported by a significant reduction in cells expressing Tgfb protein in heart tissue (*p* = 0.03), along with a non-significant reduction in cells expressing the downstream Tgfb target, pSMAD2 (*p* = 0.08, 4.5 ± 0.8 vs. 10.5 ± 2.9%) (Figure 2E–G). Furthermore, Western blot analysis of proteins also showed a significant reduction in the matrix protein alpha smooth muscle actin (aSMA, *p* = 0.02) and TGFb (*p* = 0.02) in the heart in the I/R + CIN038 group vs. the I/R + vehicle group (Figure 2H–J).

### 2.3. ERK and NFKb Protein Phosphorylation Were Attenuated by CIN038 in Cardiac Tissue and Cells

**Figure 3:**
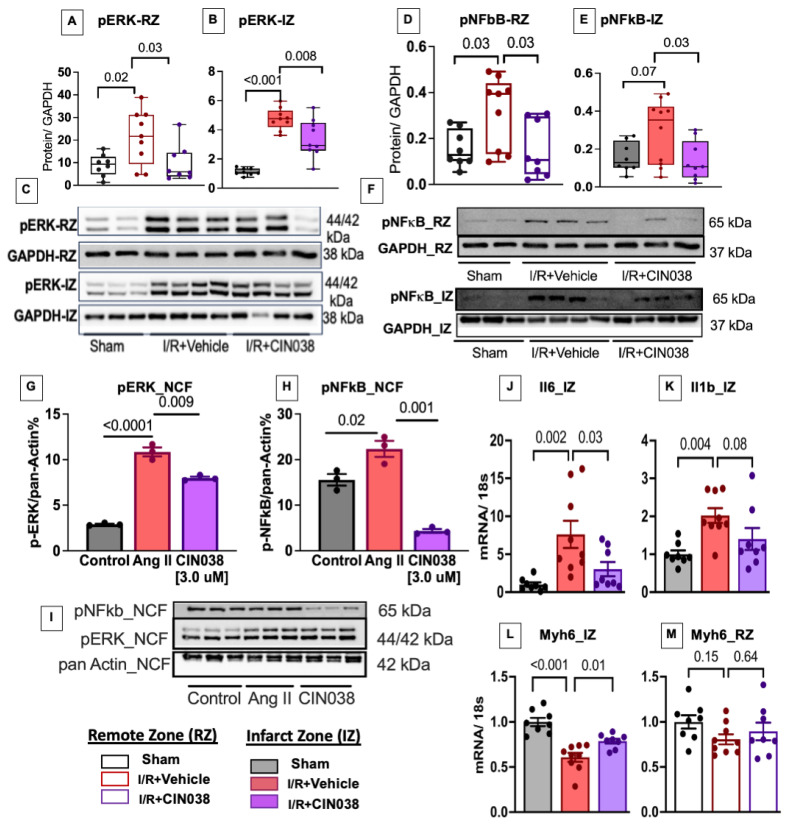
CIN038 inhibited ERK, NF-kB, and inflammatory signalling. Western blot showing expression of phosphorylated ERK-pERK in (**A**) the remote zone, RZ, and (**B**) the infarct zone, IZ, (**C**) with representative blots. Phosphorylated NFkB-pNFkB in (**D**) the RZ and (**E**) the IZ and (**F**) with representative blots. Neonatal cardiac fibroblast (NCF) expression of (**G**) pERK and (**H**) pNFkB with (**I**) representative blots, for two independent experiment replicates. qPCR results showing relative mRNA expression of (**J**–**L**) *Il6*, *Il1b*, and *Myh6* in the IZ and (**M**) *Myh6* in the RZ. Sham = 8, I/R + vehicle = 9, I/R + CIN038 = 8. Data are presented as ± SEM and analysed using one-way ANOVA with Dunnett’s post hoc test. Significance at *p* < 0.05.

We investigated the effect of CIN038 on two other pathways that promote cardiac fibrosis: the extracellular signal-regulated kinase (ERK) and nuclear factor kappa B (NFĸB) pathways. CIN038 treatment reduced phosphorylated ERK in both the RZ (pERK, *p* = 0.03) and IZ (*p* = 0.02) (Figure 3A–C), which were significantly elevated (RZ, *p* = 0.0.17; IZ, *p* < 0.001) in the I/R + vehicle group. Similarly, phosphorylated NFĸB protein expression was attenuated in the RZ (pNFĸB, *p* = 0.03) and IZ (*p* = 0.03) of infarcted hearts treated with CIN038 (Figure 3D–F). CIN038 at a concentration of 3.0 uM also attenuated these proteins in isolated primary neonatal cardiac fibroblasts (NCFs) in the presence of angiotensin II (Ang II), a primary driver of fibrosis in I/R injury hearts (Figure 3G–I).

### 2.4. Inflammatory and Hypertrophic Signalling Were Suppressed by CIN038

We evaluated the effect of CIN038 on inflammatory and hypertrophic markers that can be highly activated by I/R injury through ERK and NFkB signalling. I/R injury increased the expression of interleukin-6 (*Il6*, *p* = 0.002) and *Il1b* (*p* = 0.004) in the IZ of the I/R + vehicle group vs. sham (Figure 3J,K). CIN038 significantly suppressed *Il6* mRNA (*p* = 0.03), while the suppression of *Il1b* did not reach significance (*p* = 0.08). Furthermore, CIN038 reversed (*p* = 0.01) the mRNA expression of *Myh6*, a hallmark of I/R injury-induced remodelling (Figure 3L). This change in *Myh6* mRNA was not observed in the RZ (Figure 3M). Interestingly, CIN038 treatment did not have a significant impact on mRNA expression of the natriuretic peptides (*Nppa* and *Nppb*) in both the infarct and remote zones (Supplementary Figure S2A–D).

### 2.5. CIN038 Inhibited STAT3 Protein Phosphorylation and mRNA of SphK1, S1PR2, and Icam1 in the Remote Zone

**Figure 4:**
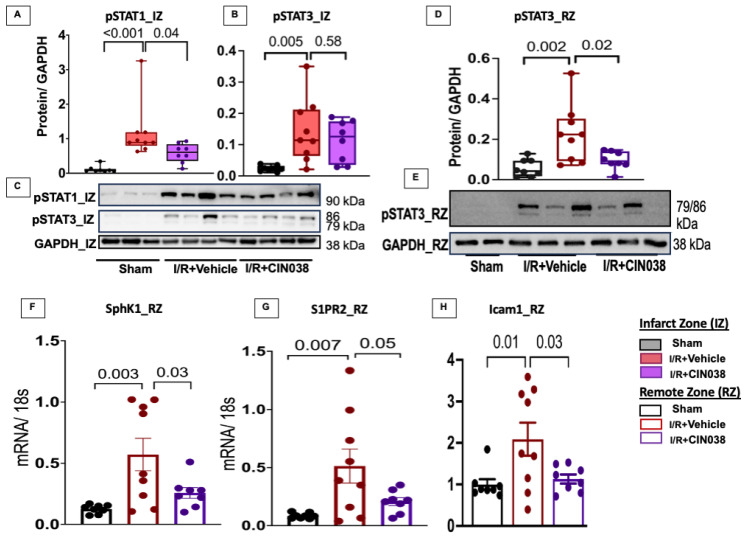
MI altered Jak/Stat pathway protein phosphorylation and mRNA levels of sphingolipid pathway-related genes. Western blot showed that I/R injury increased the expression of phosphorylated (**A**) pSTAT1 and (**B**) pSTAT3 in the infarct zone (IZ) (**C**) with representative blots. (**D**) pSTAT3 protein expression in remote zone (RZ) (**E**) with representative blots. Relative mRNA expression of sphingolipid-related genes (**F**,**G**) *Sphk1*, *S1PR2*, and (**H**) *Icam1* normalized to 18s. Sham = 8, I/R + vehicle = 9, I/R + CIN038 = 8. Data are presented as ± SEM and analysed using one-way ANOVA with Dunnett’s post hoc test. Significance at *p* < 0.05.

We then investigated the CIN038 effect on the Janus kinase/signal transducer and activator of transcription (JAK/STAT) pathway that is linked to inflammatory and hypertrophic signalling. Protein expression analysis revealed that CIN038 treatment significantly inhibited phosphorylated STAT1 (pSTAT1, *p* = 0.04) but not STAT3 (pSTAT3, *p* = 0.58) within the IZ vs. I/R + vehicle group (Figure 4A–C). Interestingly, in the RZ, pSTAT3 was significantly inhibited by CIN038 (*p* = 0.02), along with reductions in *SphK1* (*p* = 0.03) and sphingosine 1 phosphate receptor 2 (*S1pr2*, *p* = 0.05) (Figure 4D–G). The effect on *SphK1* and *SIpr2* was not evident in the IZ (Supplementary Figure S2E,F). CIN038 treatment also suppressed intercellular adhesion molecule-1 (*Icam1*, *p* = 0.03) mRNA expression in the RZ (Figure 4H). The Il6-STAT3 axis directly enhances the expression of Icam1. We also noted that I/R injury had no effect on the mRNA levels of the sphingolipid pathway nodal enzyme, dihydroceramide desaturase 1 (*Degs1*), in the heart, and these remained unchanged with CIN038 treatment (Supplementary Figure S2G,H).

### 2.6. I/R Injury-Altered Circulating CE, LPC-O, PC-P, and S1P Lipids Are Reversed by CIN038 Treatment

**Figure 5:**
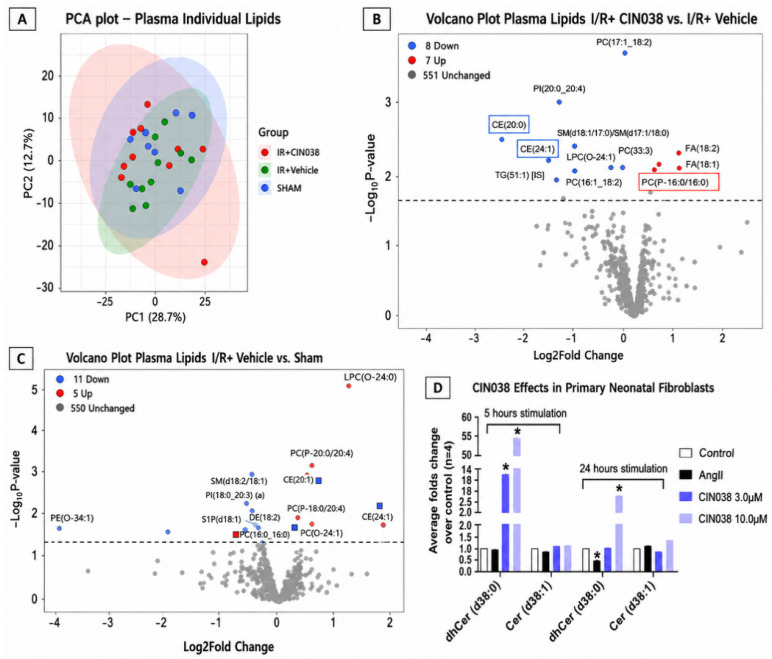
Individual plasma lipidomics and cardiac fibroblast levels of dhCer. Principal component (PCA) analysis of (**A**) individual lipid species in the sham (n = 8), I/R + vehicle (n = 9), and I/R + CIN038 (n = 8) groups. (**B**) Volcano plot showing significant changes in I/R + CIN038 vs. I/R + vehicle. An opposite reduction in I/R + vehicle vs. sham is shown in a blue rectangle, and an increase is shown in a red rectangle. Significance at *p* < 0.05 and log2FC. (**C**) Volcano plot of I/R + vehicle vs. sham showing a significant reduction in the blue circle, and the opposite change by CIN038 is indicated by the red square. Increases are shown in red circles, and the opposite change by CIN038 is indicated by blue squares. Lipid species normalised to internal standards were log2-transformed before analysis through limma in R. (**D**) Lipidomics analysis of NCFs, control (white), and incubated with Ang II (black), 3.0 µM CIN038 (blue), and 10.0 µM CIN038 (violet) at 5 and 24 h. Three independent experiments were conducted with n = 3 replicates for each time point and treatment. Data are presented as ± SEM and analysed using one-way ANOVA with Dunnett’s post hoc test, results vs. control, * *p* < 0.05.

We then examined whether CIN038 altered the levels of individual lipids in plasma. Principal component analysis (PCA) of individual plasma lipid profiles demonstrated no significant variation between groups (Figure 5A). Comparison of I/R + CIN038 plasma to I/R + vehicle showed that two lipid species of cholesteryl ester (CE), CE (20:0) and CE (24:1), and one species of lysoalkylphosphatidylcholine, LPC (O-24:0), were significantly reduced by CIN038 (Figure 5B). These were altered in the I/R + vehicle group vs. sham (blue and red squares, Figure 5C). The reduction in CE species was accompanied by increases in plasma levels of the free fatty acids (FAs), oleic (18:1) and linoleic (18:2) acid, which are precursors for CE production. The sphingomyelin (SM) species, SM (d17:1/18:0), was also increased by CIN038 treatment vs. the I/R + vehicle group. These latter individual species were not altered by I/R injury. The phosphatidylcholine P species, PC (P-16:0/16:0), was significantly reduced by CIN038 treatment, which was increased in the I/R + vehicle group.

There were no changes detected for targeted individual sphingolipid species, such as Cer and dhCer. In contrast, in cultured primary NCFs, we observed significant elevation of dhCer (d38:0) levels up to 55- and 14-fold at 5 and 24 h of treatment with CIN038, respectively (Figure 5D). Individual plasma lipid analysis showed a significant reduction in the downstream sphingolipid, sphingosine 1 phosphate (S1P), in the I/R + vehicle vs. sham group (Figure 5C). CIN038 treatment had no effect on the SIP reduction.

### 2.7. CIN038 Suppressed Pro-Inflammatory Lipids and Increased TG-O Species in the Liver

**Figure 6:**
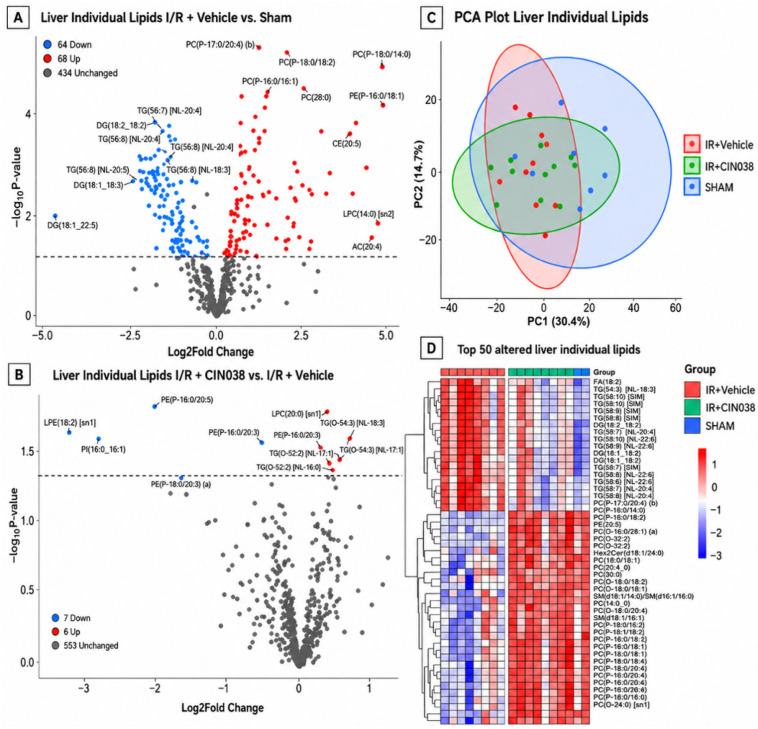
Liver individual species lipidomics. (**A**) Volcano plot of I/R + vehicle (n = 9) vs. sham (n = 8). (**B**) I/R + CIN038 (n = 8) vs. I/R + vehicle for liver individual lipidomics showing increased (red) and decreased (blue) lipids at *p* < 0.05 and log2FC. (**C**) Principal component (PCA) analysis plot and (**D**) heatmap of the top 50 lipids changed in I/R + vehicle vs. sham in the liver. Lipid species normalised to internal standards were log2-transformed before analysis through limma in R.

Next, we analysed the liver lipid profile to determine the effects of CIN038 on this major lipid-synthesising organ in the presence of I/R injury in the heart. In the I/R + vehicle group, 132 individual lipids were significantly altered out of 566 individual liver lipids analysed compared to the sham group (Figure 6A). A total of 68 of these lipid species were increased, while 64 were reduced. In contrast, CIN038 treatment had much less effect on individual liver lipid species compared to the I/R + vehicle group. There were 13 species altered by CIN038, with seven increased and six reduced. The PCA plot showed considerable overlap, with only 30.4% of the total variance explained by PC1 and only 14.7% explained by PC2 (Figure 6B,C). There were many TG species with high polyunsaturated fatty acid (PUFA) chains decreased in the I/R injury group among the top 50 altered individual species compared to the sham group; for example, TG (54:6) [NL-18:3] (Figure 6D, Supplementary Table S4). In contrast, the six TG lipid species increased by CIN038 treatment were alkyl-TGs, such as TG (O-52:2) [NL-16:0] (Figure 6B). TG-O lipids are a prominent feature of “over saturation” in the ether–lipid pathway [28]. The majority of the elevated lipid species in the top 50 lipids in the I/R injury group were members of the pro-inflammatory ether phospholipids, such as PC (P17:0/20:4) (b) and PE (P-18:1/18:1) (a) (Figure 6D). There were also elevated SM species, including SM (37:2) and SM (d18:1/14:0), hinting at increased inflammatory signalling. Interestingly, CIN038 treatment significantly reduced three ether phospholipids, which were among the seven lipid species reduced by CIN038 treatment compared to I/R + vehicle (Figure 6B). These included PE (P-16:0 (20:5), PE (P-16:0/20:3), and PE (P-18:0/20:3) (a). The reduction in LPE (18:2) [sn1], LPC (20:0 [sn1], and PI (16:0_16:1) by CIN038 treatment compared to the I/R + vehicle group shows a dampening of pro-inflammatory lipid species.

## 3. Discussion

In this study, we demonstrate that CIN038 [27] confers cardio protection following IR injury through coordinated suppression of fibrotic, inflammatory, and lipid-mediated signalling pathways. Specifically, CIN038 significantly reduced infarct size, attenuated cardiomyocyte hypertrophy, and suppressed key profibrotic pathways, including TGF-B, ERK, and NFkB signalling. These findings indicate that CIN038 is a multi-modal regulator of post-ischemic cardiac remodelling.

Recently, sphingolipids such as S1P and Cer have been implicated in CVDs [2,8,10,13]. Targeting the nodal enzyme, DES1, in the de novo sphingolipid biosynthesis pathway alters its levels [5]. Despite evidence for the beneficial effects of DES1 inhibition in cancer and insulin resistance, research in CVDs targeting DES1 has been lacking [20,29,30,31,32].

CIN038, a novel inhibitor of DES1, significantly reduced infarct size and cardiomyocyte hypertrophy, indicating a direct effect on structural remodelling processes. Cardiac remodelling alters ventricular architecture and induces phenotypic changes in myocytes and the ECM [33,34]. Infarct size is strongly correlated to remodelling and impacts cardiac function after an MI [35]. Similarly, attenuation of myocyte hypertrophy and reduction in heart weight/body weight ratio suggest that CIN038 limits compensatory hypertrophic responses. This is further supported by the reversal of *Myh6*, a key marker of cardiomyocyte contractile phenotype that is reduced during pathological remodelling [36]. Despite these structural improvements, CIN038 did not significantly restore ejection fraction or ventricular volumes. This dissociation between structural and functional recovery is well described in preclinical I/R models, where early anti-remodelling effects do not always translate to rapid improvements in systolic function [37]. It is possible that longer treatment duration or earlier intervention post-injury may be required to observe functional benefits [38]. Alternately, CIN038 may preferentially target fibrosis and inflammation rather than contractile dysfunction per se.

A key finding of this study is the robust suppression of TGFb pathway by CIN038. TGFb is a master regulator of cardiac fibrosis, driving fibroblast activation, myofibroblast differentiation, and extracellular matrix deposition [39]. CIN038 reduced *Tgfb* mRNA expression in both infarct and remote zones, with concomitant reductions in downstream targets, including *Col1a1*, *Col3a1*, and *α-SMA*. Importantly, these effects were more pronounced in the remote zone, suggesting that CIN038 may be particularly effective at limiting interstitial fibrosis and adverse remodelling distal to the infarct site. This is clinically relevant, as remote zone fibrosis contributes significantly to diastolic dysfunction and HF progression [40]. We have previously demonstrated these antifibrotic and anti-hypertrophic effects of DES1 inhibition in isolated primary NCFs and vascular cells in the presence of uremic toxins [41,42].

The reduction in TGFb protein and partial attenuation of pSMAD2 further support inhibition of canonical TGFb signalling. The incomplete suppression of SMAD phosphorylation suggests that CIN038 may also modulate non-canonical TGFb pathways, including ERK and NFkB signalling, as reflected by their significant attenuations in the remote zone and cardiac fibroblasts [43,44]. These pathways are well-established mediators of cardiac fibrosis and inflammation, acting downstream or in parallel with TGFb signalling [44]. ERK activation promotes fibroblast proliferation and collagen synthesis while NFkB drives inflammatory cytokine production and immune cell recruitment [45,46]. This is further supported by the reduction in *Il6* mRNA expression and trends toward decreased *Il1b*. Given that Il-6 can activate STAT3 and amplify fibrotic and hypertrophic responses, our findings suggests a coordinated dampening of the Il-6-STAT axis [46,47,48,49]. CIN038 exhibited region-specific effects on JAK/STAT signalling, with inhibition of pSTAT1 in the infarct zone and pSTAT3 in the remote zone. STAT3 is a critical mediator of inflammation, fibrosis, and hypertrophy in the remote myocardium [46], and its suppression aligns with the reduced *Il6* and *Icam1* mRNA expression. The cell-specific mechanisms governing this suppression warrant further exploration; for example, the macrophage-driven lipid metabolism in the expansion of cardiac fibrosis. Especially, in relation to the recent discoveries related to the protein SerpinB2 (plasminogen activator inhibitor 2) [50] linking metabolic chronic inflammation to resident macrophages, given the close crosstalk between sphingolipids, mitochondrial integrity, and downstream NF-kB signalling.

The zone-specific effects of CIN038 were also observed for the sphingolipid-related genes, *Sphk1* and *S1pr2*, which were reduced in the remote zone. These align with the observed effects of CIN038 on inflammatory targets since the Sphk1-S1P-S1PR axis is a key regulator of inflammation, fibrosis, and vascular permeability in CVD [51,52]. Interestingly, *Degs1* mRNA expression was unchanged, indicating that CIN038 does not directly modulate de novo Cer synthesis at the transcriptional level. This suggests that the observed lipid changes may occur through downstream or compartment-specific mechanisms [53].

Plasma lipidomics analysis revealed modest but targeted changes following CIN038 treatment. Reductions in CE species and LPC (O) lipids, alongside increased free fatty acids, suggest altered lipid flux and reduced esterification. Elevated CE levels are associated with CVD risk and lipid accumulation in atherosclerosis [54,55] and their reduction may reflect improved lipid handling. The increase in PC (P-16:0/16:0) with CIN038 treatment further indicates modulation of ether lipid metabolism. Ether lipids, particularly plasmalogens, are linked to oxidative stress and inflammation [56]. Changes in lipid profile after an MI occur within 24–48 h, reaching their peak within 4–7 days [57]. In this study, at 28 days post-MI, changes in lipid species in plasma were minimal compared to the pronounced changes in the liver. Consistent with this, there were no significant alterations in plasma Cer or dhCer species detected. This lack of change in plasma dhCer levels may be related to the metabolic feature of sphingolipid compartmentalisation rather than compound-related issues. This is supported by the 55-fold increase in dhCer levels at 5 h of CIN038 treatment observed in cultured NCFs. Furthermore, the marked reduction in S1P in I/R injury could imply impaired vascular integrity, often seen in mice models and patients with MI [58,59]. CIN038 did not restore this reduced S1P plasma level.

CIN038 also had a comparatively modest effect on the extensive liver lipid remodelling features, such as increased pro-inflammatory ether phospholipids and sphingomyelins, alongside reductions in PUFA-containing triglycerides. These changes are consistent with systemic inflammatory and metabolic dysregulation in cardiac injury [60]. CIN038 selectively reduced the pro-inflammatory ether lipids, including PE (P) species, while concomitantly increasing TG-O species, suggesting a coordinated shift in ether lipid pathway flux toward lipid storage or buffering mechanisms [61], which ultimately can limit oxidative stress and inflammatory signalling, protecting the heart. The effects of CIN038 on plasmalogens indicate an off-target effect that is yet to be investigated.

In conclusion, CIN038 attenuates post-ischemic cardiac remodelling by reducing infarct size, cardiomyocyte hypertrophy, and key profibrotic and inflammatory signalling pathways, such as TGFb, ERK, NFkB, and the IL6-STAT axis. CIN038 treatment primarily targeted remodelling with limited effect on functional improvement, highlighting that lipid modulation using CIN038 may be an effective therapeutic target for cardiac remodelling.

### 3.1. Key Findings

CIN038 altered certain markers of cardiac remodelling, such as

1. Reducing TGFβ, Coll1a1, and α-SMA in fibrosis;
2. Recovering αMHC gene expression in hypertrophy;
3. Attenuating STAT and NFĸB-related pathway activation in inflammation.

### 3.2. Limitations

This study was conducted as a preliminary proof-of-concept study towards understanding the effects of DES1 inhibition with a pharmacological tool compound, CIN038. The findings could be considerably different with improvements in compound pharmacokinetics. Analysing lipid profiles at different time points could have supplied more details in delineating any effects DES1 inhibition may have had on lipid dysregulation. For example, performing analysis of lipid profiles in plasma a few hours after surgery, and before commencing treatment at day four, in addition to the endpoint. Comparing CIN038 with other DES1 inhibitors on the market could also have contributed to a better understanding of its mode and mechanism of action. Additionally, a limitation to our Western blot data interpretation is the absence of total protein quantification, limiting our ability to rule out concurrent changes in total protein abundance.

## 4. Materials and Methods

### 4.1. Animal Model and Cell Details

All animal procedures were approved by the Alfred Research Alliance Animal Ethics Committee (AEC No. E/1949/2019/B) and complied with the Australian Code of Practice for the Care and Use of Animals for Scientific Purposes (NHMRC). Male C57Bl/6 mice (n = 30; 3 months old) were obtained and housed at the Alfred Research Alliance Precinct Animal Care Facility under standard laboratory conditions. Mice were randomised to myocardial ischemia–reperfusion (I/R; n = 22) or sham surgery (n = 8).

Primary NCFs were isolated from 1–2-day-old Sprague–Dawley rat pups, obtained from the Monash Animal Research Platform. All procedures were approved by the Alfred Medical Research and Education Precinct Animal Ethics Committee (E/1653/2016/M).

### 4.2. Myocardial Ischemia/Reperfusion Surgery

Mice were anesthetized with a ketamine (100 mg/kg), xylazine (20 mg/kg), and atropine (1.2 mg/kg) cocktail. Meloxicam and bupivacaine (0.5 mg/kg) were administered subcutaneously prior to incision. A left thoracotomy was performed via the fourth intercostal space to expose the left anterior descending (LAD) coronary artery using a surgical microscope, as previously described [62,63]. The LAD was ligated using a 7-0 silk suture with a slipknot and a releasing ring to induce regional ischemia for 40 min, followed by reperfusion. Sham-operated mice underwent identical procedures without LAD ligation.

Four days post-surgery, I/R injury mice were randomised to receive vehicle (Trappsol^®^, Cyclotherapeutics; 50 mg/kg/day, n = 11) or CIN038 (50 mg/kg/day, n = 11) for 28 days.

### 4.3. Echocardiography

Left ventricular (LV) structure and function were assessed on day 28 post-surgery. Mice were anesthetised with isoflurane (4.5% induction, 1.7% maintenance) and kept at 37 °C. Transthoracic echocardiography was performed using a Vevo2100 imaging system (FUJIFILM VisualSonics, Toronto, ON, Canada) equipped with a 40 MHz linear array transducer. Parasternal long-axis B-mode images were acquired and analysed offline using Vevo LAB software (v3.2.5) by an observer blinded to treatment groups.

### 4.4. Cardiac Hemodynamic Assessment

Mice were anesthetised with isoflurane and placed in a supine position. A 1.4 Fr Millar pressure–volume catheter was advanced into the LV via the right carotid artery and connected to a PowerLab acquisition system (AD Instruments, Dunedin, New Zealand) at 10 kHz. Hemodynamic assessment was performed as described previously [64]. Cardiac performance was assessed by stroke work (SW), end-diastolic and end-systolic volumes (Ved, Ves), maximal LV pressure rise (dP/dt_max), and end-systolic pressure (Pes). Load-independent systolic and diastolic function were evaluated using ESPVR and EDPVR. Diastolic function was quantified by end-diastolic pressure (Ped), isovolumetric relaxation constant (τ), and dP/dt_min. Additional parameters included LV developed pressure (P_dev), maximal and minimal LV pressures (P_max, P_min), and arterial elastance (Ea). Two mice (one per group) were excluded due to incomplete data acquisition.

### 4.5. Plasma and Tissue Collection

Following hemodynamic assessment, mice were euthanised by cardiac puncture. A blood sample was centrifuged (1800 rpm, 5 min) to isolate plasma, which was snap-frozen and stored at −80 °C. Tissue weights were recorded at necropsy. The LV was dissected into base, mid-section, and apex. The mid-section was subdivided into infarct and remote zones; samples were snap-frozen or processed for histology as indicated.

### 4.6. Lipidomic Analysis of the Plasma and Liver

Plasma and liver samples from mice were processed for liquid chromatography–mass spectrometry (LC-MS) according to previously published methods [65]. The liver was chosen to determine whether CIN038 affected liver lipid synthesis. Liver tissue (~16.2 ± 1.8 mg) was homogenised and sonicated on ice. Lipids were extracted from 10 µL plasma or liver homogenate and analysed using an Agilent 6490 QQQ mass spectrometer coupled to an Agilent 1290 HPLC system with a ZORBAX Eclipse Plus C18 column (2.1 × 100 mm, 1.8 μm, Agilent, Santa Clara, CA, USA). Dynamic scheduled multiple reaction monitoring (MRM) was performed in positive ion mode. Lipid species were quantified using internal standards and MassHunter software (B.07.00). Lipids were assigned to a specific lipid species based on MRM (precursor/product) ion pairs and retention time. The ratio of each analyte peak to the corresponding internal standard was used to quantify the lipids. MRM values for lipidomics can be provided upon request.

Lipid class abundance data were analysed in R (v4.5.2) using the limma package (v 3.66.2). Following prior normalisation to internal standards, data were log2-transformed before analysis. Differential abundance testing was performed using linear models with empirical Bayes moderation. Statistical significance was defined as *p* < 0.05. PCA was used to assess global variation in lipid profiles between groups.

### 4.7. Histochemical and Immunohistochemical Analysis

Mid-LV tissue sections were fixed in 10% neutral-buffered formalin, paraffin-embedded, sectioned (4 µm), and stained with PSR to assess fibrosis [16]. Infarct size was quantified as the mean percentage of endocardial and epicardial scar circumference. An example is provided in Supplementary Figure S1. Cardiomyocyte hypertrophy was assessed from H&E-stained sections by measuring the cross-sectional area of 100 randomly selected myocytes per animal by 2 blinded investigators. Only the myocytes with transverse orientation within the remote zone were included.

Immunohistochemistry was performed for TGFb (Ab25121, Abcam, Cambridge, UK) and phosphorylated SMAD2 (PA5-105000, Invitrogen, Waltham, MA, USA). Signal detection was performed using diaminobenzidine as previously described [66], and quantification was performed using Aperio ImageScope (Leica Biosystems, Nussloch, Germany) by a blinded observer.

### 4.8. Cardiac Tissue Protein Expression Analysis Through Western Blot

LV infarct and remote zone tissues were homogenised in lysis buffer containing protease and phosphatase inhibitors. Protein expression of STAT1/3 (8826/9145), ERK (4376), NFκB (3033), and total proteins for TGFβ (3709) and α-SMA (Ab5694, Abcam) were assessed by SDS-PAGE and immunoblotted as previously described [15]. All antibodies with catalogue numbers only were purchased from Cell Signalling Technologies (Danvers, MA, USA). Protein bands were detected using Super Signal West Pico Chemiluminescence substrate (Thermo Fisher Scientific, Rockford, IL, USA) and analysed using Image Lab software v6.1 (BioRad Laboratories, Hercules, CA, USA). Proteins were normalised to glyceraldehyde 3-phosphate dehydrogenase (GAPDH) or β-actin.

### 4.9. Quantitative PCR

Total RNA was isolated using TRIzol and reverse-transcribed using a high-capacity reverse transcription kit (Thermofisher, Waltham, MA, USA). Quantitative PCR was performed as described previously [67]. Genes related to fibrosis; *Tgfb1*, *Col1a1*, and *Col3aI*, hypertrophy; *Myh6*, *Nppa* and *Nppb*, inflammation; *Tnfa*, *Il1b* and *Il6*, and sphingolipid; *S1pr1-3*, were analysed. The enzymes *Sk1* and *Degs1*, were also quantified. *18s* rRNA was used as the endogenous control. Primer sequences are provided in Supplementary Table S1.

### 4.10. Neonatal Cardiac Fibroblast Isolation and Culture

Primary ventricular NCFs were isolated from 1–2-day-old Sprague–Dawley rat pups using collagenase digestion, as previously described [68,69]. Hearts were excised, rinsed in cold PBS, and ventricles were cut into 3–5 mm pieces. Tissue was digested in collagenase I (1:25; Sigma-Aldrich, St. Louis, MO, USA) in 1× Ads buffer with gentle agitation at 10 min intervals. Following digestion, cell suspensions were layered onto a Percoll gradient and centrifuged at 3300 rpm for 30 min. Fibroblasts (upper layer) were collected, washed in 1× Ads buffer, and resuspended in DMEM containing 10% newborn calf serum (pH 7.2). NCFs were maintained in DMEM with 10% fetal bovine serum (FBS) and used at passage 2. For experiments, NCFs were detached using diluted trypsin–EDTA, reseeded at the required densities, and maintained in DMEM with 10% FBS for 24 h prior to treatment with CIN038. Lipidomics analysis was performed as described previously.

### 4.11. Data Analysis

Data are presented as mean ± standard error of the mean (SEM). Statistical analyses were performed using Student’s *t*-test or one-way ANOVA with Dunnett’s correction, as appropriate. Analyses were conducted in GraphPad Prism 9. Lipidomic analyses were corrected for multiple comparisons using the Benjamini–Hochberg false discovery rate in R. A two-tailed *p* < 0.05 was considered statistically significant.

## Data Availability

The original contributions presented in this study are included in the article/Supplementary Materials. Further inquiries can be directed to the corresponding author.

## Supplementary Materials

The following supporting information can be downloaded at: https://www.mdpi.com/article/10.3390/ijms27135776/s1.

## Abbreviations

Cer	Ceramide
Degs1	Delta 4-desaturase, sphingolipid 1 gene
DES1	Dihydroceramide desaturase 1 enzyme
DhCer	Dihydroceramide
DhS1P	Dihydrosphingosine 1 phosphate
HF	Heart failure
I/R	Ischemia–reperfusion
KXA	Ketamine/xylazine/atropine
LAD	Left anterior descending artery
NCFs	Neonatal cardiac fibroblasts
PEDS1	Plasmanylethanolamine desaturase 1
PV Loop	Pressure–volume loop
S1P	Sphingosine 1 phosphate
S1pr2	Sphingosine 1 phosphate receptor 2
TGs	Triglycerides
