# Supplementary material for: Suppression of Post-Ischemic Cardiac Remodelling and Inflammatory Response by a Novel Sphingolipid Modifier, CIN038
Source: Int J Mol Sci. 2026 Jun 26;27(13):5776. doi: 10.3390/ijms27135776 (PMC13361308; doi:10.3390/ijms27135776)
Supplement: Supplementary file 1 [file ijms-27-05776-s001.zip › ijms-4342697-Supplementary Figures.pdf]

Supplementary Figures

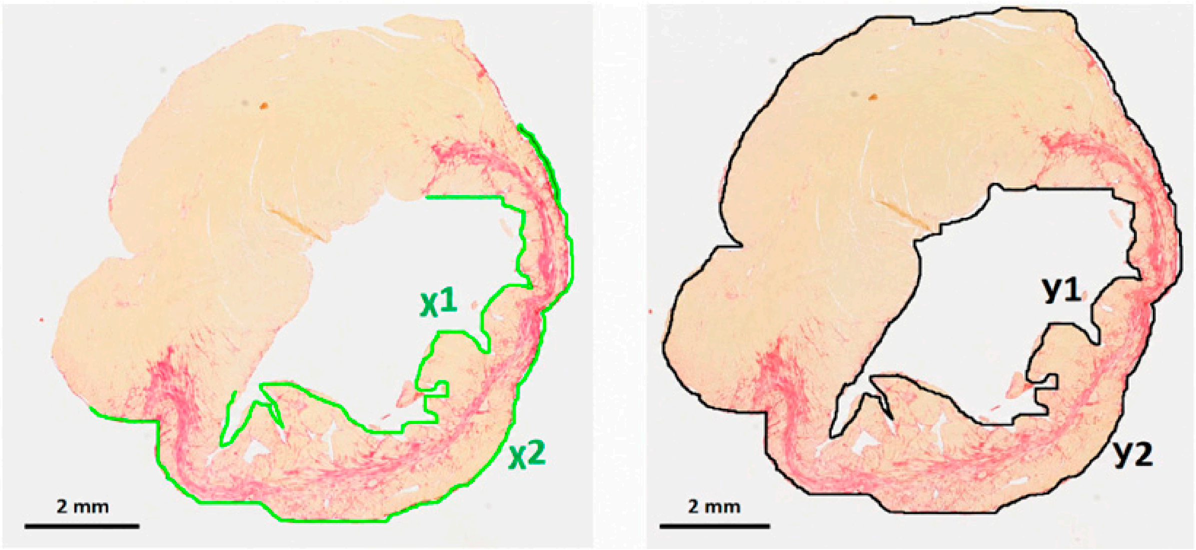

Supplementary Figure S1. Infarct size measurement illustration. Infarct size was measured using the following formula: Infarct size (%) = [(x1/y1) + (x2/y2)] / 2 *100, where x1: infarct length on the endocardium (μM), y1: total length of endocardium (μM), x2: infarct length on the epicardium (μM), y2: total length of epicardium (μM). 20 X magnification.

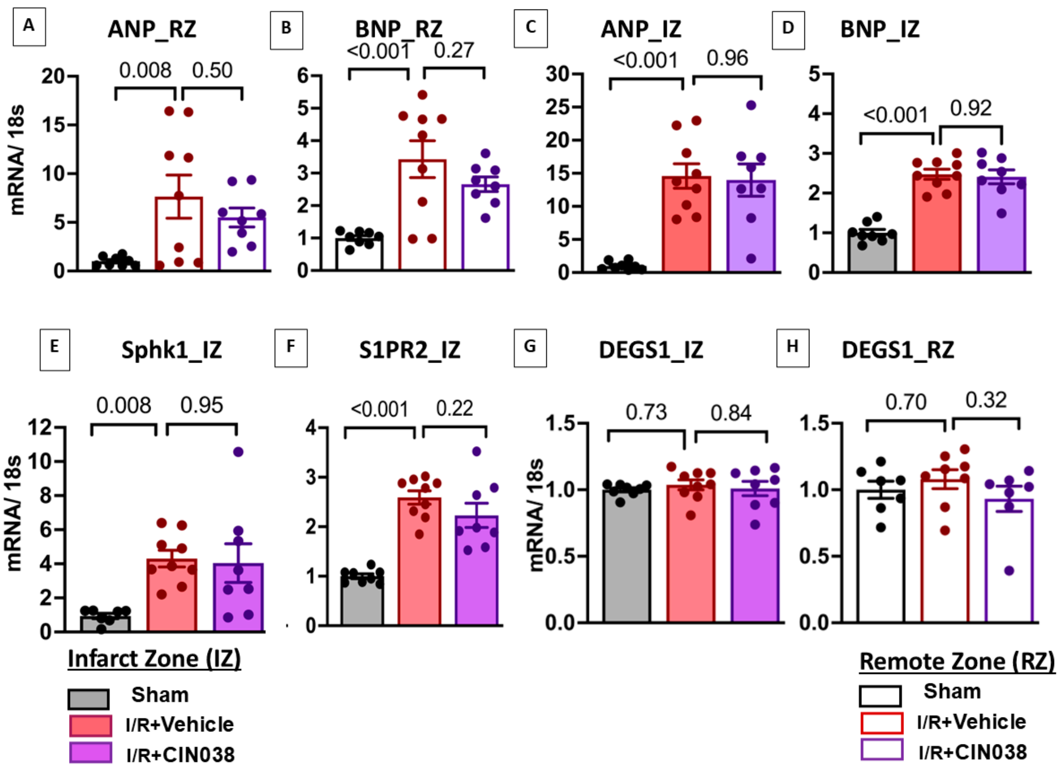

Supplementary Figure S2. CIN038 had no effect on hypertrophic and sphingolipid targets in cardiac tissue. 4 weeks daily IP injections of CIN038 treatment (n=8) at 50 mg/kg in C57Bl/6 mice had no significant effect on **A-B**) ANP and BNP mRNA in remote zone (RZ) and **C-D**) ANP and BNP in infarct zone (IZ) compared to I/R + vehicle group (n=9). **E-H)** SphK1, S1PR2, Degs1 in IZ and RZ were not affected by CIN038 treatment. Relative mRNA levels normalized to 18s. Sham=8. Data are presented as ± SEM and analysed using One way ANOVA with Dunnett's post hoc test. Significance at $p<0.05$.
