# Supplementary material for: Suppression of Post-Ischemic Cardiac Remodelling and Inflammatory Response by a Novel Sphingolipid Modifier, CIN038
Source: Int J Mol Sci. 2026 Jun 26;27(13):5776. doi: 10.3390/ijms27135776 (PMC13361308; doi:10.3390/ijms27135776)
Supplement: Supplementary file 1 [file ijms-27-05776-s001.zip › ijms-4342697-Supplementary File S1- Western Blot Originals.pdf]

Supplementary File S1

Western Blot Original Blots

Figure 2

-aSMA & GAPDH

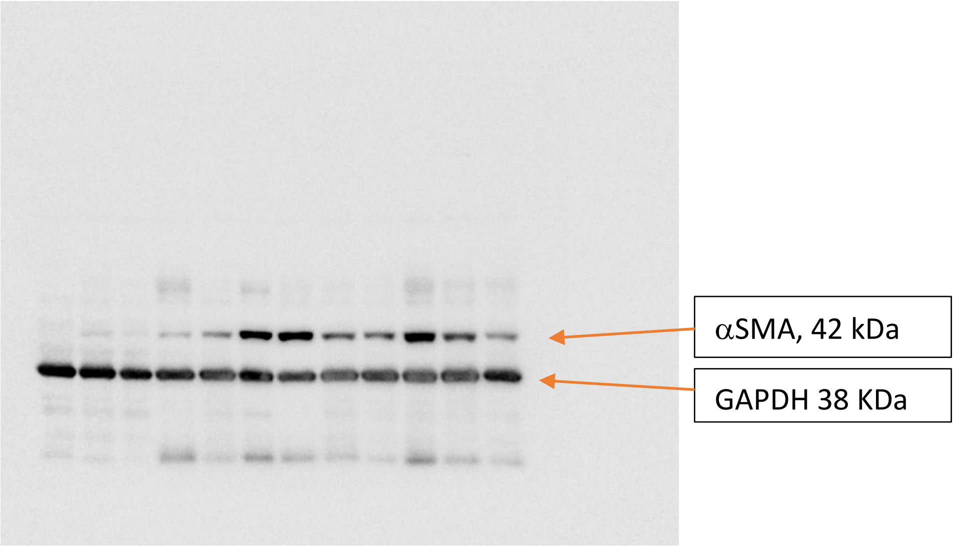

- TGFb

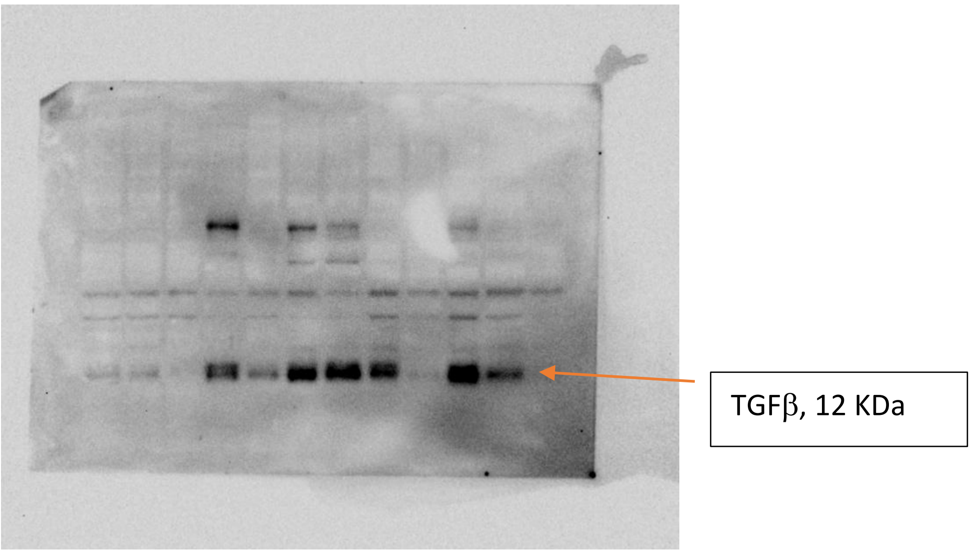

GAPDH

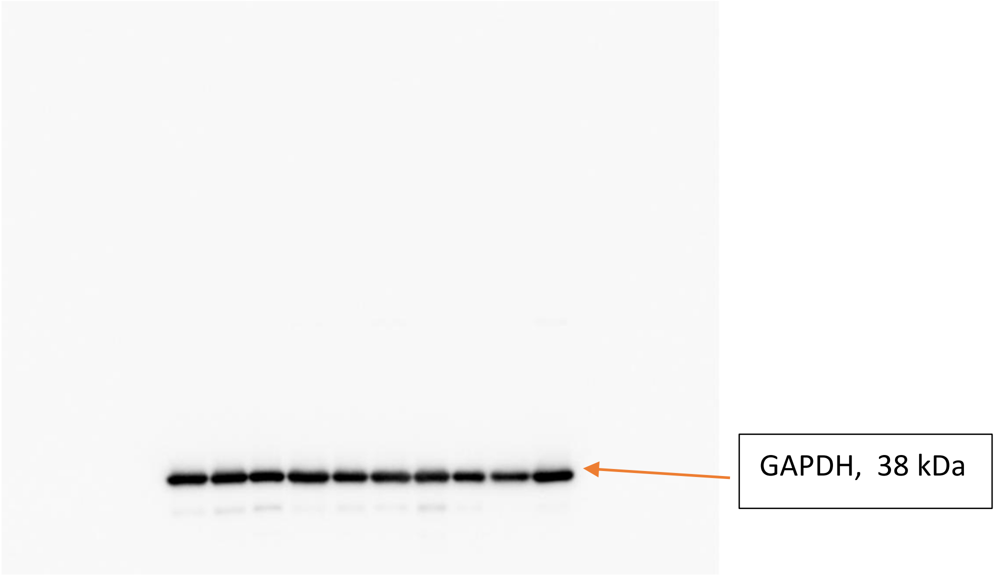

Figure 3

- GAPDH- (Infarct Zone)

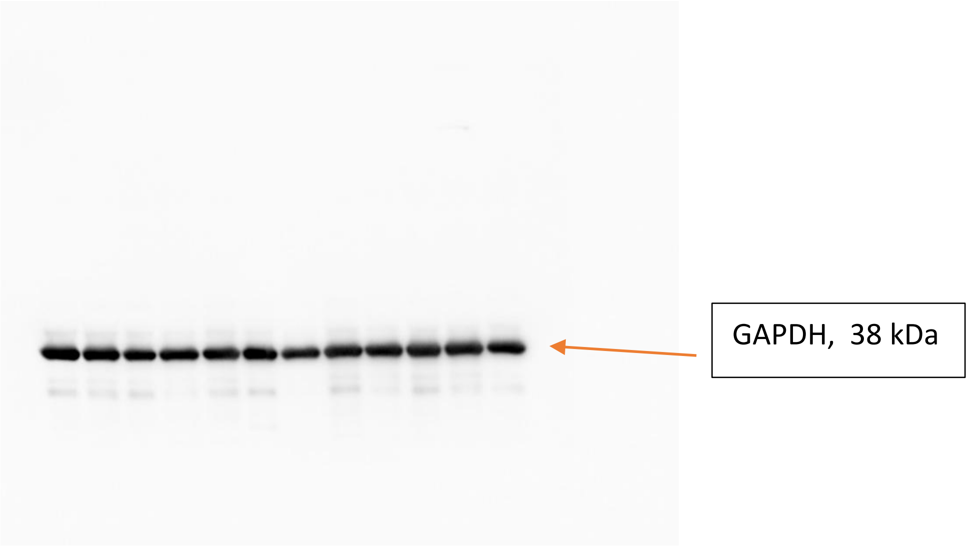

B- pERK (Infarct Zone)

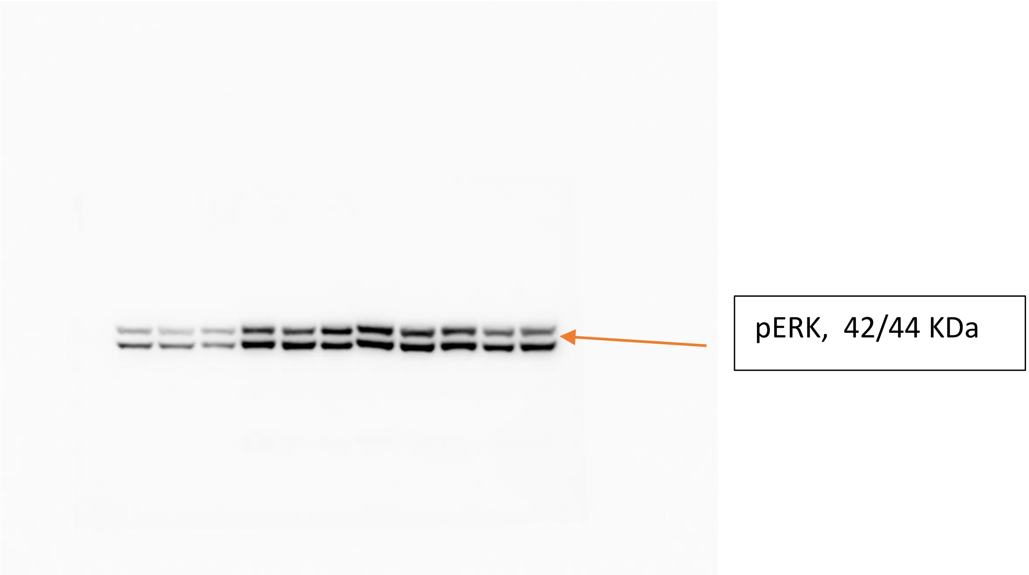

B-pERK (Remote Zone)

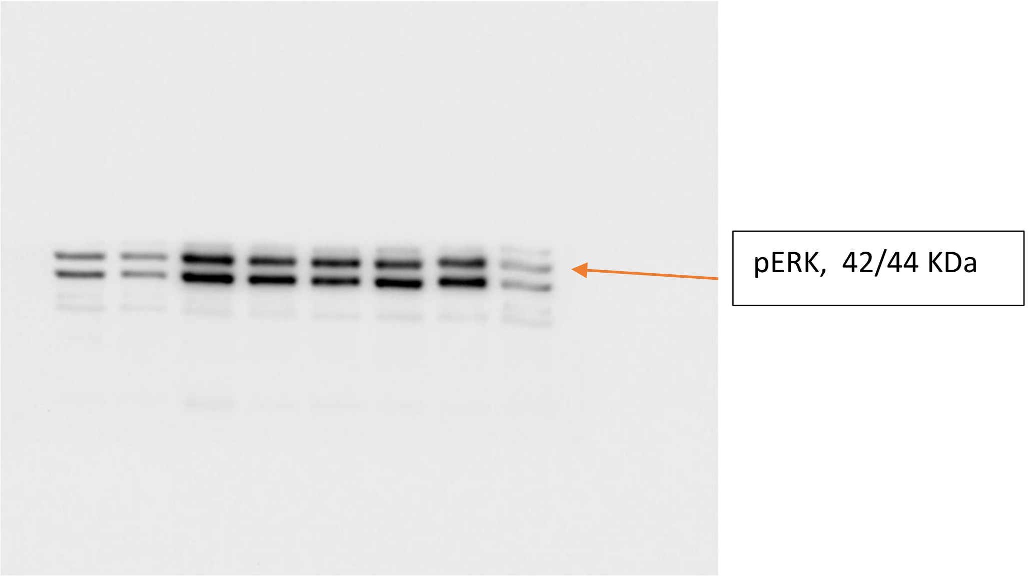

GAPDH

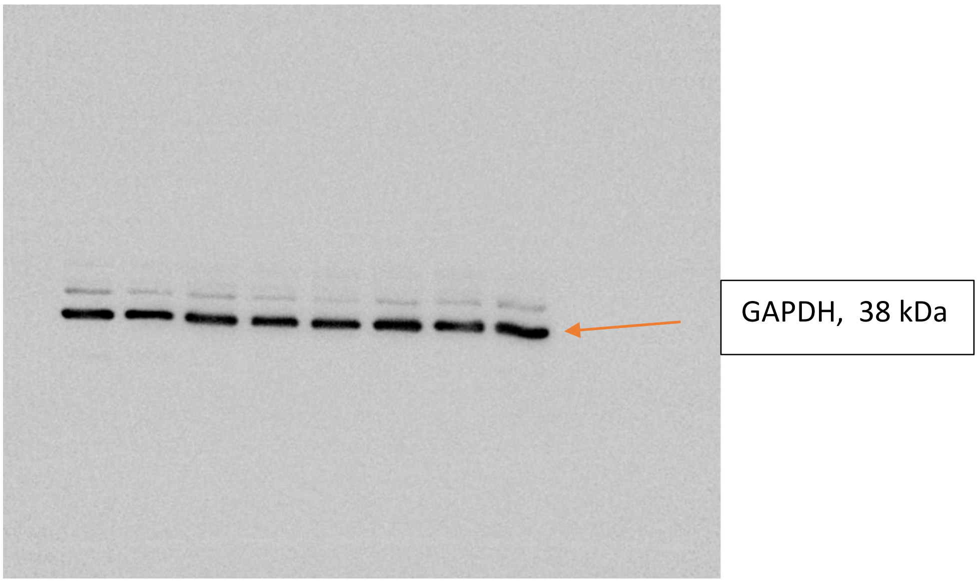

Figure 4

p-STAT1 (Infarct Zone)

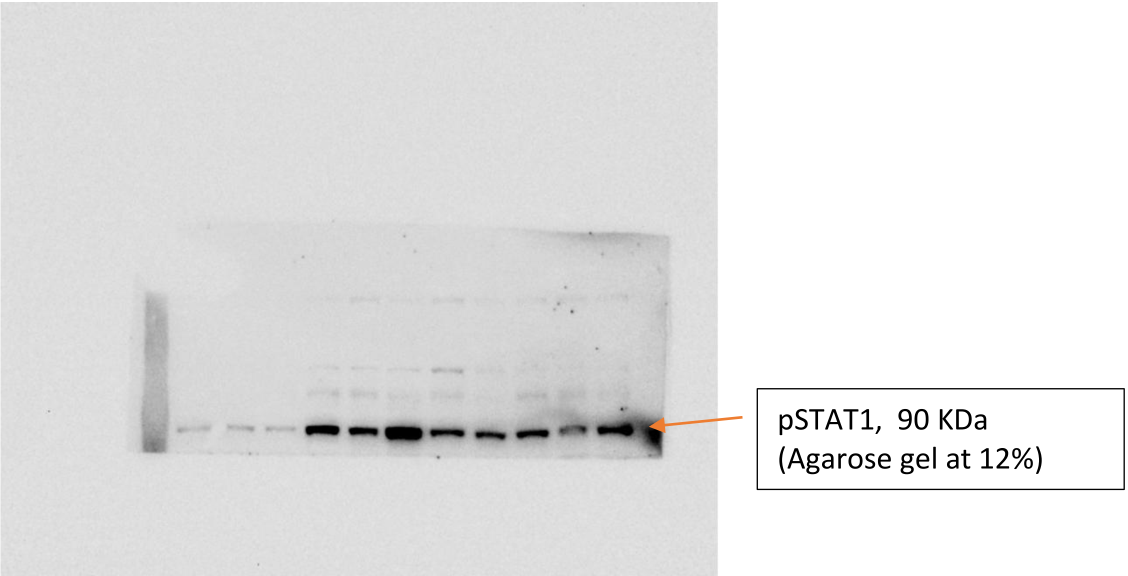

p-STAT3 (Infarct Zone)

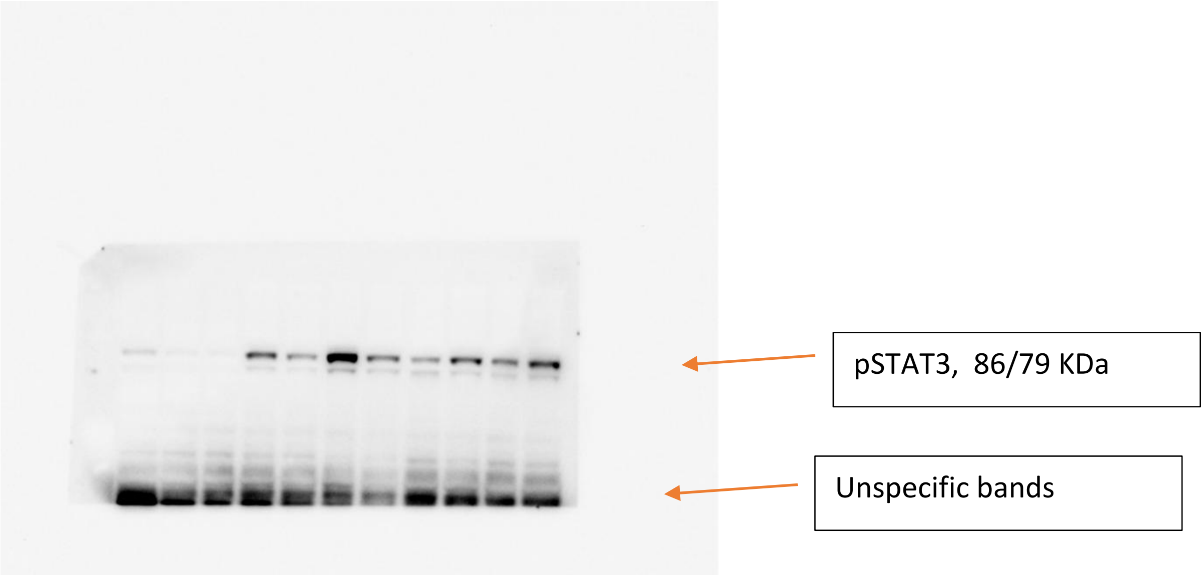

GAPDH (Infarct Zone)

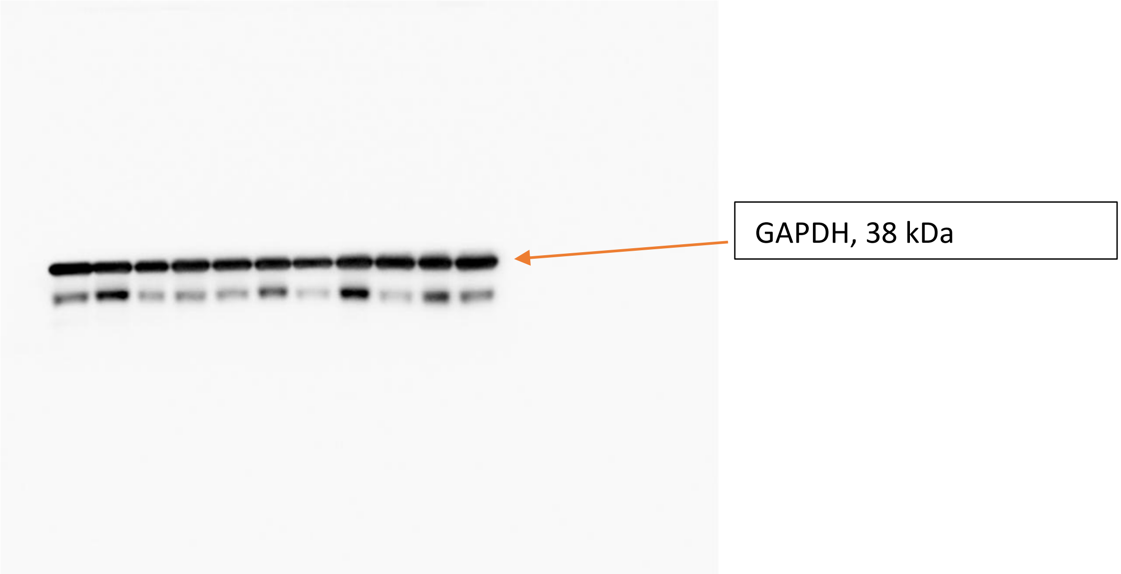

Figure 3E- NFκB (Infarct Zone)

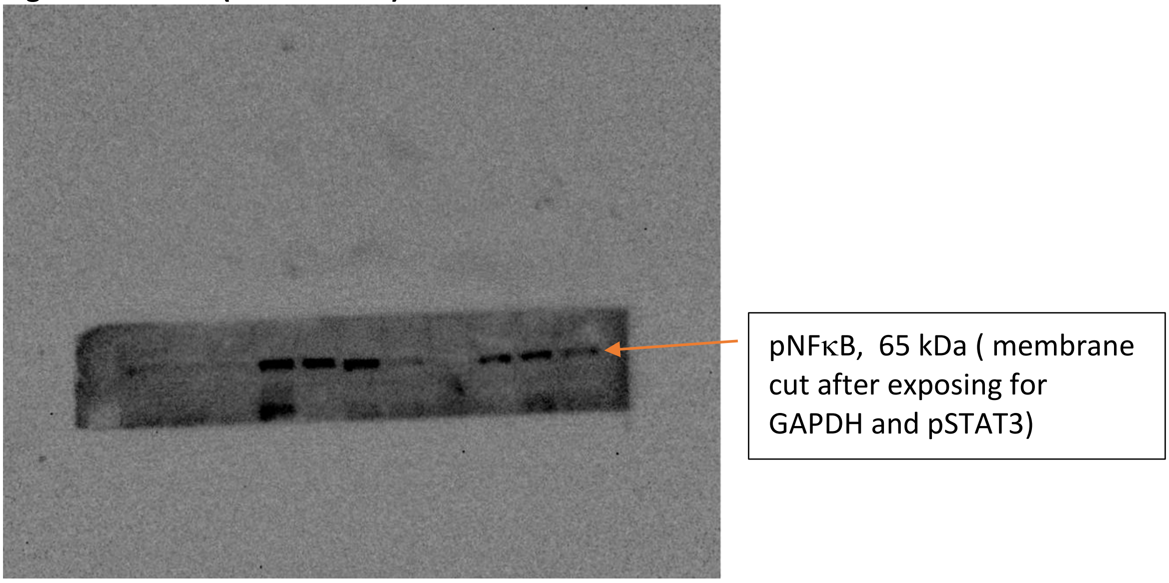

GAPDH (Before exposing for NFκB)

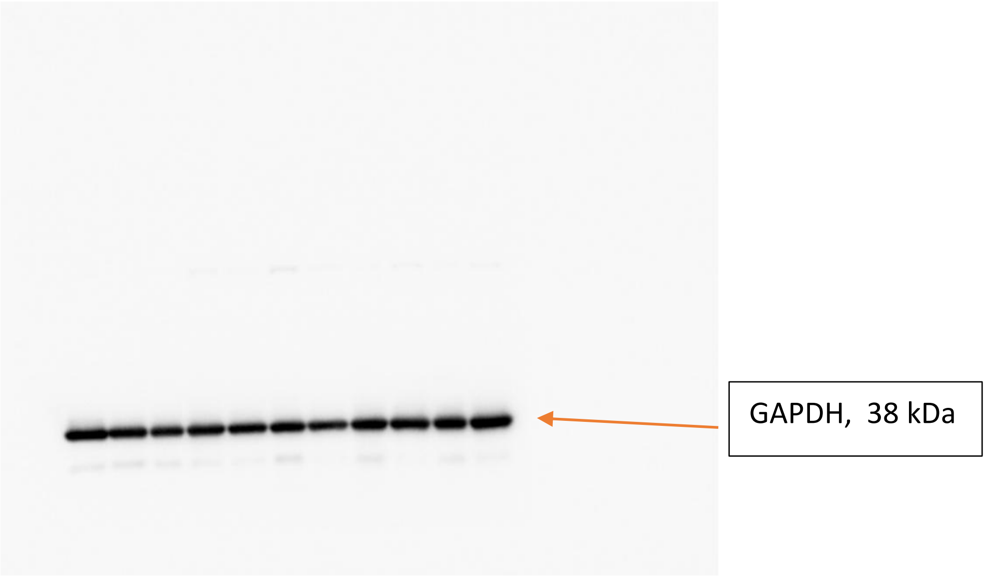

4D-STAT3 & 3D-NFκB (Remote Zone)

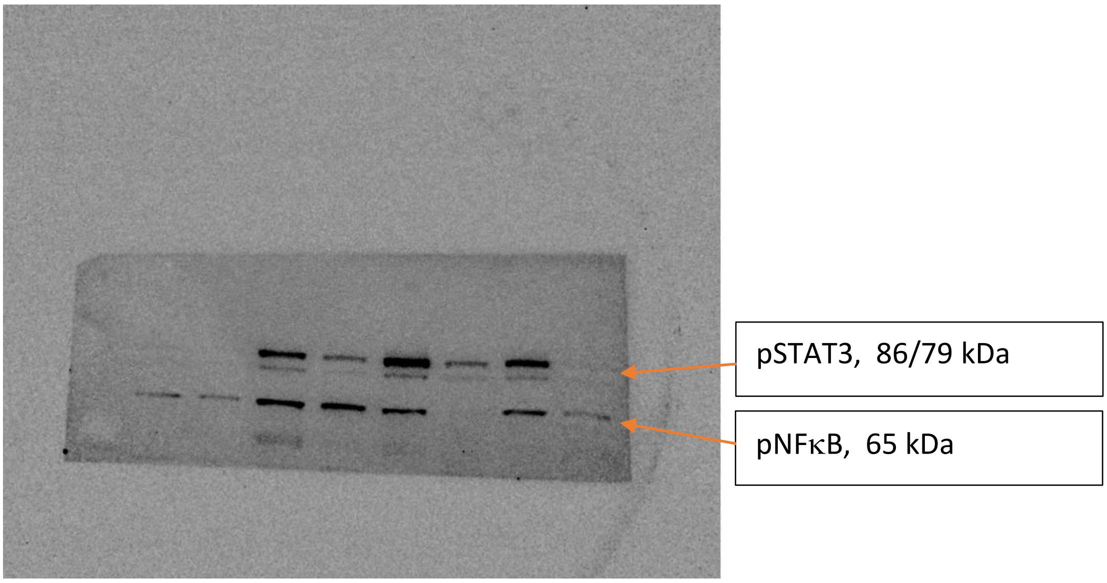

GAPDH

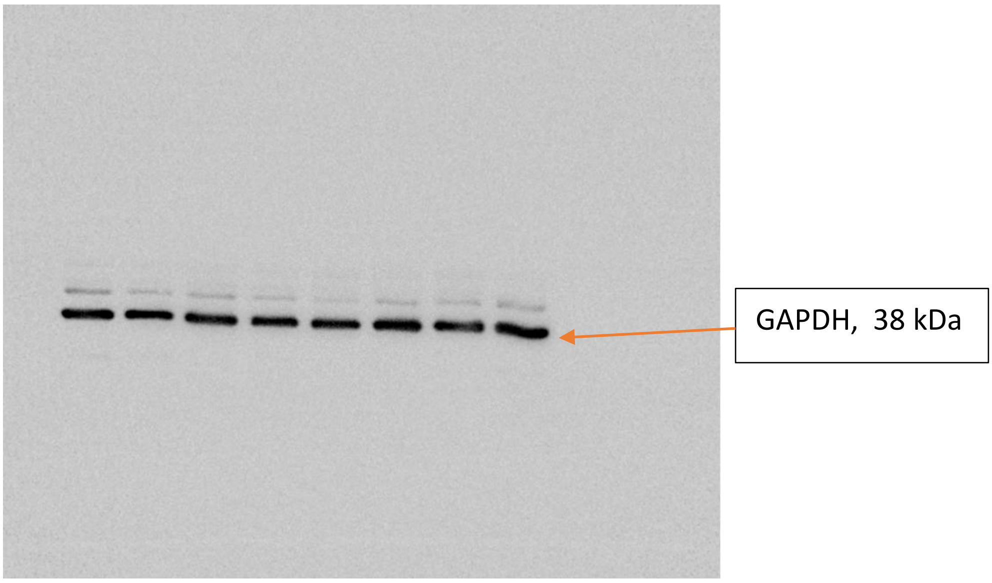

Figure 3G- Cell Culture Western Blot

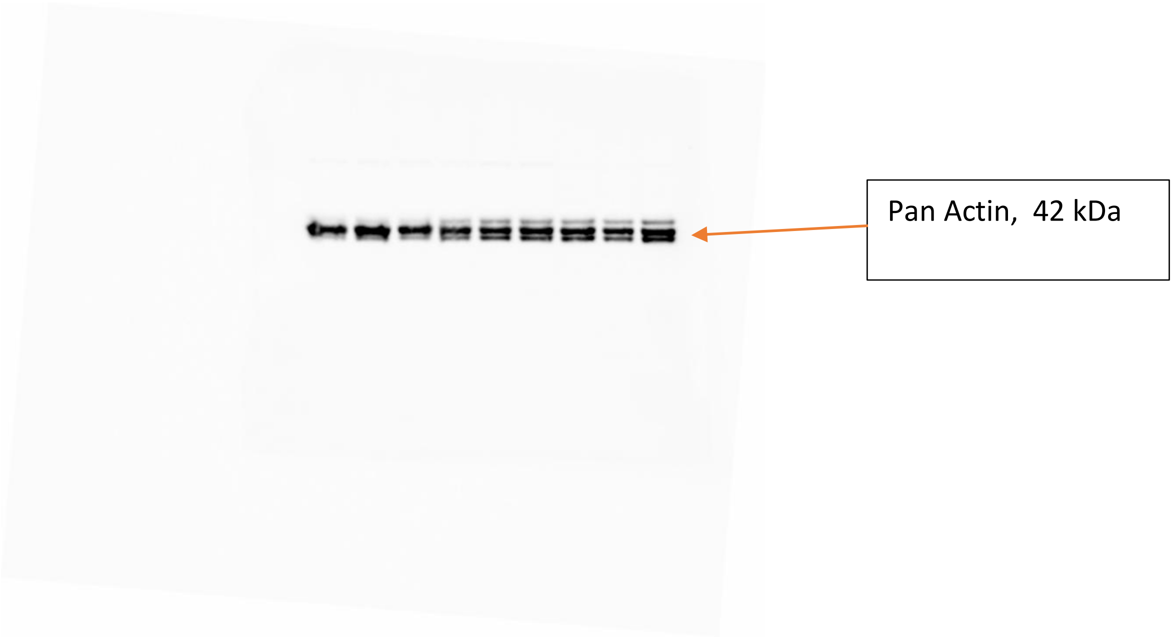

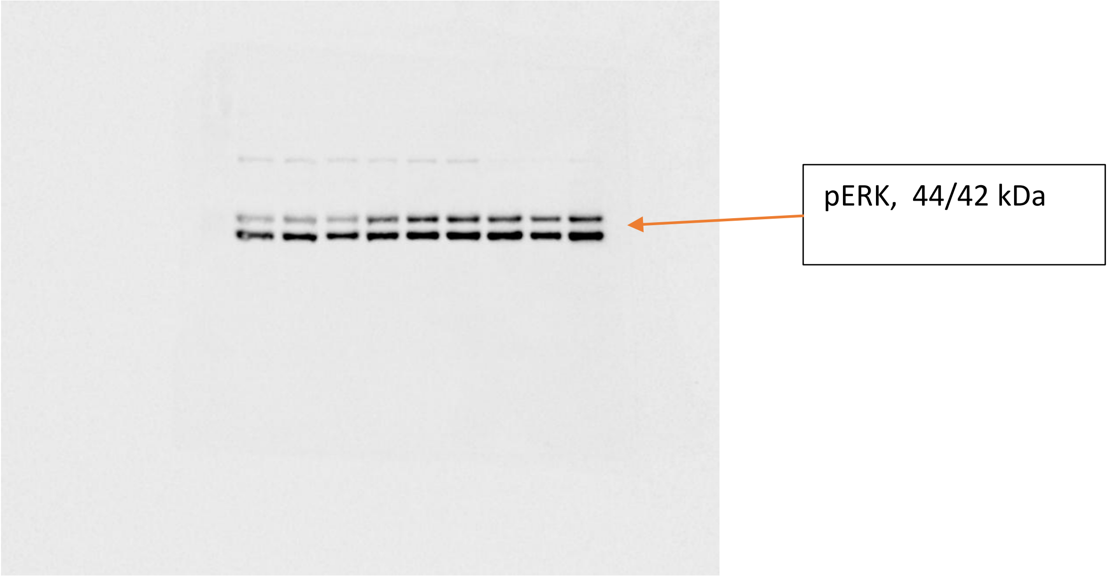
pERK, 44/42 kDa

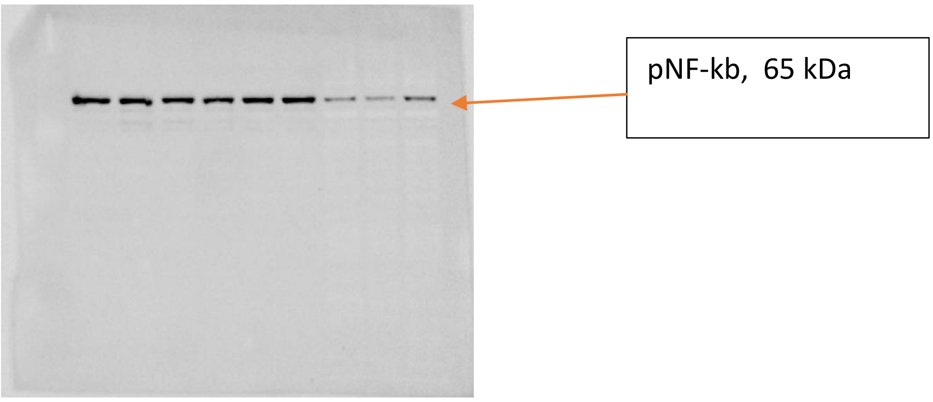
pNF-kb, 65 kDa
