# Supplementary material for: Suppression of Post-Ischemic Cardiac Remodelling and Inflammatory Response by a Novel Sphingolipid Modifier, CIN038
Source: Int J Mol Sci. 2026 Jun 26;27(13):5776. doi: 10.3390/ijms27135776 (PMC13361308; doi:10.3390/ijms27135776)
Supplement: Supplementary file 1 [file ijms-27-05776-s001.zip › ijms-4342697-Supplementary Table S1.pdf]

Supplementary Table S1. List of mouse primers and their sequences

Gene	Primer Sequence	Efficiency, R^2	Slope
TGFβ1	*Forward: 5’-CCA GCC GCG GGA CTC-3’*<br>*Reverse: 5’-TTC CGT TTC ACC AGC TCC AT-3’*	82%, 0.99	-3.8
CTGF	*Forward: 5’-GCG GCG AGT CCT TCC AA-3’*<br>*Reverse: 5’-CCA CGG CCC CAT CCA-3’*	84%, 0.99	-3.7
TIMP1	*Forward: 5’- GTA AAG ACC TAT AGT GCT GGC TG- 3’*<br>*Reverse: 5’-GAG CAT CTG ATC TGT CCA CAA-3’*	74%, 0.99	-4.1
TIMP2	*Forward: 5’ GGTCACAGAGAAGAGCATCAATG 3’*<br>*Reverse: 5’-GGT CAC AGA GAA GAG CAT CAA TG-3’*	71%, 0.97	-4.1
Coll 1a1	*Forward: 5’-TGG ATT CCA GTT CGA GAG TAT G-3’*<br>*Reverse: 5’-AGT GAT AGG TGA TGT TCT GG- 3’*	81%, 0.99	-3.3
Coll 3a1	*Forward: 5’-ACT CAA GAG TGG AGAATA CTG-3’*<br>*Reverse: 5’-AAC ATG TTT CTT CTC TGC AC-3’*	80%, 0.99	-3.9
MMP2	*Forward: 5’-GGG TCC ATT CTG CCA GCA CTC- 3’*<br>*Reverse: 5’-CTC CAG AAC TTG TCT CCT GCA A- 3’*	74%, 0.99	-4.0
MMP9	*Forward: 5’-CTT CCA GTA CCA AGA CAA AG-3’*<br>*Reverse:5’-ACC TTG TTC ACC TCA TTT TG-3’*	69%, 0.94	-3.2
*αMHC	*Forward: 5’-TTG GCA CGG ACT GCG TCA TC- 3’*<br>*Reverse: 5’- GAG CCT CAA GAG TTT GCT GAA- 3’*	87%, 0.99	-3.5
*ANP	*Forward: 5’-ATC TGA TGG ATT TCA AGA ACC- 3’*	73%, 0.99	-4.1

	Reverse: 5’-CTC TGA GAC GGG TTG ACT TC- 3’		
*BNP	*Forward: 5’-ACA ATC CAC GAT GCA GAA GCT-3’*	72%, 0.99	-4.3
	Reverse: 5’-GGG CCT TGG TCC TTT GAG A- 3’		
TNFα	*Forward: 5’-CTA TGT CTC AGC CTC TTC TC- 3’*	86, 0.86	-3.4
	Reverse: 5’-CAT TTG GGA ACTTCT CAT CC- 3’		
IL-1β	*Forward: 5’-GGA TGA TGA TGA TAA CCT GC- 3’*	83%, 0.97	-3.9
	Reverse: 5’-CAT GGA GAA TATCAC TTG TTG G- 3’		
IL-6	*Forward: 5’-AAG AAA TGA TGG ATG CTA CC- 3’*	68%, 0.95	-3.2
	Reverse: 5’-GAG TTT CTG TAT CTC TCT GAA G- 3’		
*S1PR1	*Forward: 5’-CAT GAG GTG AAA TGT GAG AG-3’*	82%, 0.99	-4.0
	Reverse: 5’-AGT TGG TTG AAA TGG ATC AC-3’		
*S1PR2	*Forward: 5’-ATC CTG TCA TCT ATA CTG G G-3’*	65%, 0.97	-4.5
	Reverse: 5’-CAG AAA TGT CGG TGATGT AG-3’		
*S1PR3	*Forward: 5’-GAA CGA GAG CCT ATT TTC AAC- 3’*	72%, 0.98	-4.1
	Reverse: 5’-TCC TAG AGA CAG ATG GTT AC- 3’		
*DEGS1	*Forward: 5’-ATC TTA GCG AAG TAT CCA GAG- 3’*	68%, 0.99	-4.1
	Reverse: 5’-CAG AGT CAT GGA ATG GTT AAG- 3’		
*SK1	*Forward: 5’-CTT TAA ACT GAT GCT CAC CG- 3’*	77%, 0.93	-3.4
	Reverse: 5’-TAC ATA GGG GTT TCT GGA TG- 3’		

*18s	*Forward: 5’-TCG AGG CCC TGT AAT TGG AA- 3’*<br>*Reverse: 5’ -CCC TCC AAT TGG ACC TCG TT- 3’*	94%, 0.99	-3.6
*GAPDH	*Reverse: 5’-AGC CCA GGA TGC CCT TTA GT- 3’*<br><br>*Forward: 5’-GAC ATG CCG CCT GGA GAA AC- 3’*	80%, 0.98	-3.9

* *Sigma,* St. Louis, MO, USA
