# Supplementary material for: Suppression of Post-Ischemic Cardiac Remodelling and Inflammatory Response by a Novel Sphingolipid Modifier, CIN038
Source: Int J Mol Sci. 2026 Jun 26;27(13):5776. doi: 10.3390/ijms27135776 (PMC13361308; doi:10.3390/ijms27135776)
Supplement: Supplementary file 1 [file ijms-27-05776-s001.zip › ijms-4342697-Supplementary Table S2.pdf]

Supplementary Table S2. I/R surgery induced changes in echocardiographic parameters did not change with CIN038 treatment. Echocardiographic quantification of cardiac function parameters at endpoint (D27). Sham n= 8, I/R+Vehicle n= 9, I/R+CIN038 n=8. 1-way ANOVA results for sham vs. I/R+Vehicle and I/R+Vehicle vs. I/R+CIN038 are as shown in the table.

**Parameters**	**Sham**	**I/R+Vehicle**		**I/R+CIN038**	
**(Units)**	**Mean ± SEM**	**Mean ± SEM**	***p* Value**	**Mean ± SEM**	***p* Value**
HR (bpm)	466.8±16.1	486.4±11.9	0.33	503.4±11.9	0.32
LVAs (mm)	14.4±0.44	22.1±1.8	0.002*	19.9±1.5	0.35
LVAd (mm)	22.3±0.73	27.3±1.9	0.032*	25.8±1.38	0.47
LVVs (uL)	26.4±1.4	64.2±9.4	0.0009*	56.7±7.0	0.31
LVVd (uL)	56.2±3	95.6±30.9	0.002*	75.01±16.1	0.11
SV (uL)	29.8±1.7	24.2±1.9	0.04*	25.8±1.3	0.28
EF (%)	52.9±0.8	29.5±3.3	<0.0001*	35.3±9.2	0.035*
CO (ml/min)	13.9±0.9	11.3±0.9	0.07	12.9±0.6	0.17

Heart rate (HR), left ventricle systolic area (LVAs), LV diastolic area (LVAd), LV systolic volume (LVVs), LV diastolic volume (LVVd), stroke volume (SV), ejection fraction (EF) and cardiac output (CO).
