# Supplementary material for: Suppression of Post-Ischemic Cardiac Remodelling and Inflammatory Response by a Novel Sphingolipid Modifier, CIN038
Source: Int J Mol Sci. 2026 Jun 26;27(13):5776. doi: 10.3390/ijms27135776 (PMC13361308; doi:10.3390/ijms27135776)
Supplement: Supplementary file 1 [file ijms-27-05776-s001.zip › ijms-4342697-Supplementary Table S3.pdf]

Supplementary Table S3. CIN038 treatment had no effect on I/R injury induced changes in hemodynamic parameters. Quantification of pressure volume (PV) loop analysis of hemodynamic parameters at day 28. Sham n= 8, I/R+Vehicle= 9, I/R+CIN038=9, two mice were excluded, due to incomplete measurements. 1-way ANOVA results for sham vs I/R+Vehicle and I/R+Vehicle vs. I/R+CIN038 are as shown in the table.

PV Loop Parameters	Sham	I/R+Vehicle		I/R+CIN038	
	**Mean ±SEM**	**Mean ±SEM**	**vs. Sham *p*-Value**	**Mean ±SEM**	**vs. I/R+Veh *p*- Value**
SW (mmHg*µL)	1893.7±190.9	1553.3± 156.3	0.2	1604.9± 101.6	0.7
ESPVR (mmHg/ml)	2.8±0.5	1.8±0.2	0.08	1.4±0.2	0.2
EDPVR ((mmHg/ml)	0.4±0.06	0.5±0.05	0.7	0.3±0.07	0.3
Ves (µL)	23.1±4.3	51.4±15.5	0.1	44.5±7.9	0.6
Ved (µL)	42.5±6.6	71.5±15.7	0.1	64.0±7.0	0.6
Pmax (mmHg)	98.2±2.4	85.3±2.2	0.001*	84.6±1.8	0.8
Pmin (mmHg)	-0.0001± 0.00008	0.03±0.03	0.3	0.00006± 0.00009	0.3
Pdev (mmHg)	98.2±2.4	85.3±2.3	0.001*	84.2±1.8	0.8
Pes (mmHg)	93.5±2.1	81.6±2.6	0.003*	81.5±1.7	0.9
Ped (mmHg)	5.2±0.4	5.3±0.5	0.8	6.1±0.7	0.3
Ea (mmHg/µL)	4.4±0.7	3.6±0.4	0.4	3.4±0.3	0.7
dP/dt max (mmHg/s)	10771.8± 766.6	7543.8± 398.2	0.002*	7975.4± 508.9	0.5
dP/dt min (mmHg/s)	-9523.00± 690.5	-5881.9± 328.1	0.0001*	-6172.1± 353.3	0.5
τ (ms)	5.5±0.1	7.0±0.2	0.0001*	6.7±0.3	0.4

Stroke work (SW), end systolic pressure-volume relationship (ESPVR), end diastolic pressure-volume relationship (ESPVR), end systolic volume (esv), end diastolic volume (edv), maximum pressure (Pmax), minimum pressure (Pmin), developed pressure (Pdev), ventricular end systolic pressure (Pes), ventricular end diastolic pressure (Ped), Arterial elastance (Ea), maximum and minimum left ventricular pressure changes (dP/dt max, dP/dt min), isovolumetric relaxation constant (τ).
