# Supplementary figures and images for: Suppression of Post-Ischemic Cardiac Remodelling and Inflammatory Response by a Novel Sphingolipid Modifier, CIN038
Source: Int J Mol Sci. 2026 Jun 26;27(13):5776. doi: 10.3390/ijms27135776 (PMC13361308; doi:10.3390/ijms27135776)

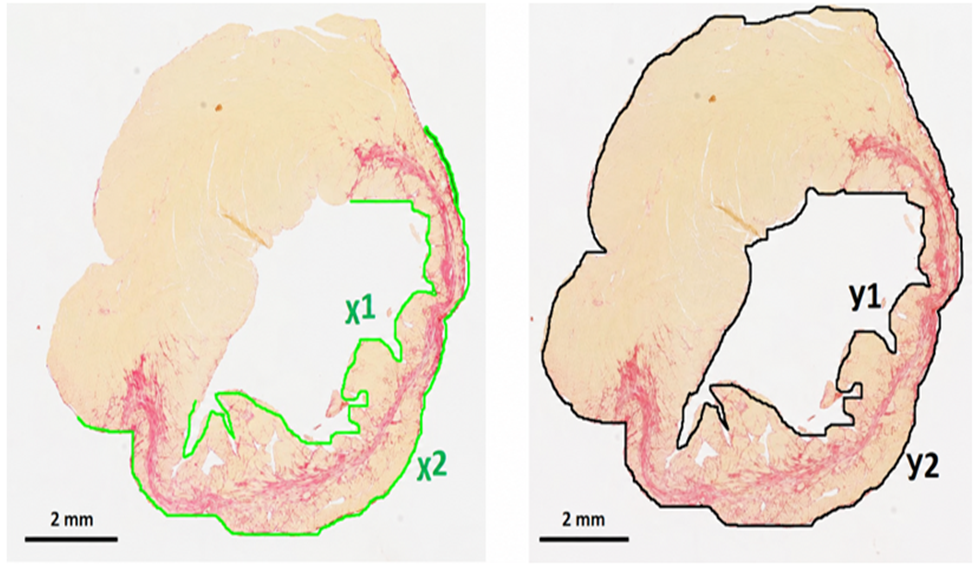

Supplement: Supplementary file 1 [file ijms-27-05776-s001.zip › ijms-4342697-Supplementary Figure S1.tif]

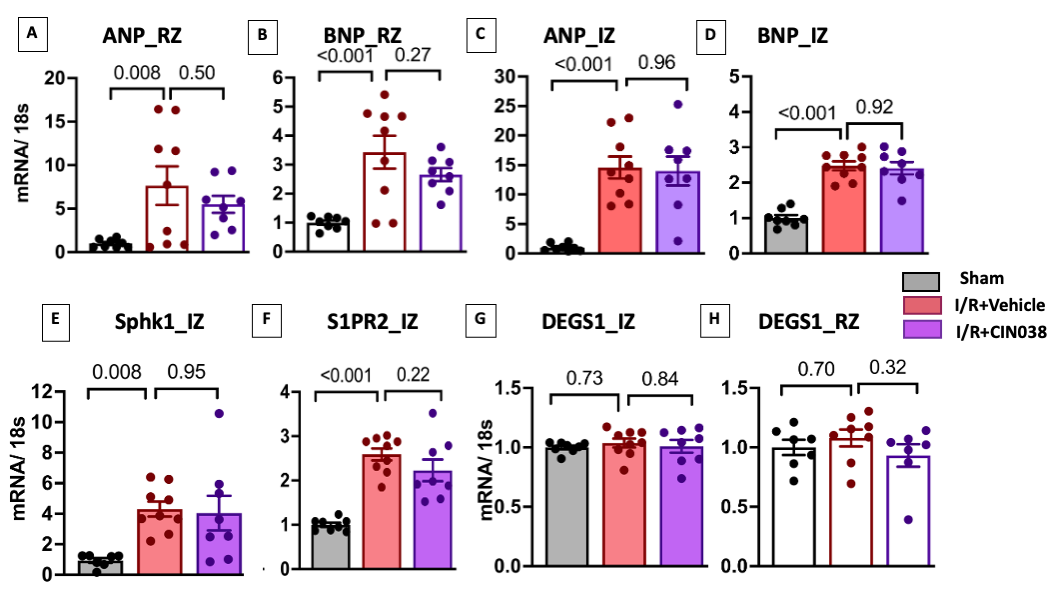

Supplement: Supplementary file 1 [file ijms-27-05776-s001.zip › ijms-4342697-Supplementary Figure S2.tiff]
